# The Extent of Universal Health Coverage for Maternal Health Services in Eastern Uganda: A Cross Sectional Study

**DOI:** 10.1007/s10995-021-03357-3

**Published:** 2021-12-30

**Authors:** Clara Lindberg, Tryphena Nareeba, Dan Kajungu, Atsumi Hirose

**Affiliations:** 1 Department of Learning, Informatics, Management and Ethics, Karolinska Institute, 171 77 Stockholm, Sweden; 2 Demographic Surveillance Site, Centre for Public Health and Population Research, Makerere University, New Mulago Hill Road, Mulago, Kampala, Uganda; 3 Centre for Health and Population Research, Makerere University, New Mulago Hill Road, Mulago, Kampala, Uganda; 4 Iganga/Mayuge Health and Demographic Surveillance Site, Kampala, Uganda; 5 School of Public Health, Imperial College London, London, UK; 6 Department of Global Public Health, Karolinska Institute, 171 77 Stockholm, Sweden

**Keywords:** Maternal health, Universal health coverage, Health service coverage, Effective coverage, Financial coverage

## Abstract

**Objective:**

Monitoring essential health services coverage is important to inform resource allocation for the attainment of the Sustainable Development Goal 3. The objective was to assess service, effective and financial coverages of maternal healthcare services and their equity, using health and demographic surveillance site data in eastern Uganda.

**Methods:**

Between Nov 2018 and Feb 2019, 638 resident women giving birth in 2017 were surveyed. Among them, 386 were randomly sampled in a follow-up survey (Feb 2019) on pregnancy and delivery payments and contents of care. Service coverage (antenatal care visits, skilled birth attendance, institutional delivery and one postnatal visit), effective coverage (antenatal and postnatal care content) and financial coverage (out-of-pocket payments for antenatal and delivery care and health insurance coverage) were measured, stratified by socio-economic status, education level and place of residence.

**Results:**

Coverage of skilled birth attendance and institutional delivery was both high (88%), while coverage of postnatal visit was low (51%). Effective antenatal care was lower than effective postnatal care (38% vs 76%). Financial coverage was low: 91% of women made out-of-pocket payments for delivery services. Equity analysis showed coverage of institutional delivery was higher for wealthier and peri-urban women and these women made higher out-of-pocket payments. In contrast, coverage of a postnatal visit was higher for rural women and poorest women.

**Conclusion:**

Maternal health coverage in eastern Uganda is not universal and particularly low for postnatal visit, effective antenatal care and financial coverage. Analysing healthcare payments and quality by healthcare provider sector is potential future research.

**Supplementary Information:**

The online version contains supplementary material available at 10.1007/s10995-021-03357-3.

## Significance Statement

Universal coverage of maternal health services is essential in reducing maternal morbidity and mortality. Measurements of universal health coverage should capture service, quality, financial and equity coverage.

We exploited Health and Demographic Surveillance Site data to measure universal health coverage, which provides an opportunity to track progress at district level. Coverage measurements indicate gaps in postnatal care service and in antenatal- and postnatal care quality. Postnatal care service coverage was higher among women from poorer and rural households, while quality indicators for postnatal care were higher among women from wealthier and urban households. Women with higher education, more wealth and urban residency make higher out-of-pocket payments for delivery service, warranting further research into contents of care.

## Introduction

Universal Health Coverage (UHC) has gained momentum as a key global health system goal, since the formulation in the world health assembly in 2005 (World Health Assembly, 2005, May 25). It is included in the Sustainable Development Goals (SDG) Agenda outlined in target 3.8 (World Health Organization, 2019). UHC means that everyone has access to the quality healthcare services they need without suffering financial hardship and includes service coverage, financial coverage and population coverage (World Health Organization, 2010). Universal coverage of quality intra-partum care services, such as Emergency Obstetric Care (EmOC) and delivery with a skilled birth attendant (SBA) are fundamental to reduce maternal mortality because most maternal deaths are preventable with timely and high-quality care (Campbell & Graham, 2006).

Low-income countries have a disproportionately high burden of maternal mortality and UHC plays a key role in achieving the global target of reducing the maternal mortality ratio to less than 70 per 100,000 live births (World Health Organization, 2019, September). A lack of reduction in maternal mortality despite increased service coverage during the decade of the Millennium Development Goals period has highlighted the importance of service quality (Koblinsky et al., 2016). Financial coverage is crucial, as women with obstetric complications can be exposed to catastrophic expenditure (Kruk et al., 2016). Addressing urban–rural and socio-economic inequities in coverage is essential to effectively reduce maternal mortality (Ruhago et al., 2012).

Uganda aims to achieve universal health coverage by improving risk-pooling through national health insurance and community health financing mechanisms (Ministry of Health, 2010, July). The ongoing five-year Supporting Policy Engagement for Evidence-based Decisions initiative supports and influences health policy and systems changes to advance UHC in Uganda (Makerere University School of Public Health, 2019).

In 1999, The Ministry of Health established a package of essential health services known as the Ugandan National Minimum Health Care Package (Ministry of Health, 1999). It details the minimum health services provided for free by the public (and to some extent Private-Not-For-Profit) providers to the population (Ssengooba, 2004), including the maternal health service package of antenatal care (ANC), routine maternity services, and EmOC (Ministry of Health, 1999). However, sub-standard delivery of the Minimum Health Care Package has been reported due to insufficient funding, inadequate planning and cost estimations of included health interventions, such as ANC services (Ssengooba, 2004). Informal payments at public facilities occur (Xu et al., 2006), despite the official abolishment of user-fees in 2001 (Nabyonga Orem et al., 2011), indicating that universal provision of essential maternal health services is questionable.

Quantifying maternal health service coverage is vital for local policy formulation and decision-making about resource allocation, particularly with Uganda’s current efforts to progress the health system towards UHC. The aim of this study was therefore to measure UHC for essential maternal health services and to assess equity for these services.

## Methods

### Study Setting and Data Collection

This study used cross-sectional data collected at the Iganga Mayuge Health and Demographic Surveillance Site (IMHDSS) in eastern Uganda (Kajungu et al., 2020). Founded in 2005, the IMHDSS population cohort covers 65 villages in a clearly demarcated area within Iganga and Mayuge districts. Since its inception, all residents in the area have been followed up at biannual or annual censuses during which information about births, deaths and migration are recorded. Currently there are 90,568 residents from 18,634 households, with 60% living in rural and 40% in peri-urban areas. There are 23 health facilities within and at the border of the Demographic Surveillance Area, including one public hospital, 15 level II Health Centres (HC) (eleven public, three Private-Not-For-Profit and one Private-For-Profit), six level III HCs (five public and one Private-Not-For-Profit), one level IV HC (public) and two private clinics.

The data consisted of (1) census data on sociodemographic information, antenatal, intrapartum and postnatal service use from the latest two rounds conducted between April and July 2017 and November 2017 and May 2018 respectively, (2) data collected on financial coverage during an SDG survey (n = 5500 households) between November 2018 and February 2019, and (3) data collected on antenatal and postnatal services and out-of-pocket payments (hereafter referred to as the pregnancy and delivery survey) (n = 449 women) (Online Resource 1 and 2). Data from the three sources were linked using unique individual IMHDSS identifiers. All surveys were conducted in both the local language Lusoga and English by trained interviewers from the two districts. Census data was collected using paper forms while the SDG, and pregnancy and delivery surveys were electronically collected using tablets.

### Study Population and Sample

For the SDG survey, a stratified random sampling method was applied to capture 100 households from each of the 65 villages within IMHDSS. This resulted in a sample of 5,500 households, when villages with less than 100 households were merged. Included in the study were 638 women who gave birth in 2017. For the pregnancy and delivery survey, a sample size of 385 was calculated assuming a significance level of 0.05 with one degree of freedom (*Z-value*), a margin of error (*d*) of 0.05 (Charan & Biswas, 2013) and an expected postnatal care coverage of 0.50 in the population (*p*), the lowest service coverage indicator (Demographic & Health Survey program, 2016), to obtain a conservative estimate.

$$ Sample\,  size = Z_{{1 - \alpha /2^{2} }} \times p \times \frac{{\left( {1 - p} \right)}}{{d^{2} }} $$

$$ Sample\, size = 1.96^{2} \times 0.50 \times \frac{{\left( {1 - 0.50} \right)}}{{0.05^{2} }} = 384.16 $$

To ensure data was retrieved from 385 women, 449 women were randomly sampled.

### Outcome Variables

Essential maternal health service coverage, healthcare quality, financial protection, and equity coverage were assessed, in line with the World Health Organisation/ World Bank (WHO/WB) framework for measuring UHC (World Health Organization and World Bank, 2014).

To assess the extent of maternal health service coverage, four indicators were chosen: At least four ANC visits, SBA, institutional delivery and one postnatal visit. SBA refers to births attended by a nurse, midwife or doctor and institutional delivery is births delivered in a hospital, clinic or health centre. One postnatal visit by a Village Health Team (VHT) was included to account for the postpartum period of maternal health (World Health Organization, 2013a, 2013b).

To assess healthcare quality, we adopted the WHO/WB recommended effective coverage approach which measures the content of care received (Ng et al., 2014). For ANC quality, we included blood pressure and weight measurements, urine and blood samples taken at least once, tetanus vaccination, and information about pregnancy danger signs from a health worker (World Health Organization, 2016). For postnatal care (PNC) quality, we included Polio and Tuberculosis (TB) vaccination given to the newborn and the woman receiving a post-delivery health check by a nurse, midwife or doctor (World Health Organization, 2013a, 2013b, October). We defined full coverage of the ANC and PNC quality indicators as effective ANC and effective PNC coverage respectively.

To measure the extent of financial coverage, three indicators were used: the extent of out-of-pocket payments for ANC, out-of-pocket payments for delivery service and health insurance coverage for maternal health services (World Health Organization, 2013a, 2013b; World Health Organization and World Bank, 2014).

### Covariates for Equity Analysis

To assess the equity of each service coverage, socio-economic status (SES), level of education and place of residence were used. SES was measured by the household wealth index developed at IMHDSS, based on household assets as outlined and used by the Uganda Bureau of Statistics and reported in Waiswa, et al. (2015). Included household asset items were re-categorized into binary categories before conducting principal component analysis (Vyas & Kumaranayake, 2006) in the statistical software programme Stata ver. 14 for Windows.

Education was measured as the highest level of education attained. Place of residence was classified as peri-urban if it was densely populated, the majority of households had piped water and the main activity was trading. If the area was scarcely populated, the majority of households didn’t have piped water and the main activity was farming, it was classified as rural.

### Statistical Analyses

Differences between categorical outcome variables were assessed using the non-parametric Chi-square (χ^2^) test of independence, and for the ordinal variables, the Chi-square (χ^2^) test of the linear-by-linear association. Fisher’s exact tests were applied where cross-tables had any cells with expected minimum count < 5. The assumption of normality was violated for the numerical variable OOP payments (p-value < 0.05; Shapiro–Wilk test), and therefore the non-parametric Mann–Whitney U test was applied. Spearman rank correlation was applied to assess the association between numerical and ordinal variables after fulfilment of a monotonic relationship had been ascertained. Where this assumption was violated, the Kruskal–Wallis H test was applied. Data was stored in Microsoft Excel 2016 for Mac and statistical analyses were conducted in SPSS ver. 24 for Mac.

### Missing Data

Sensitivity analysis was performed to compare women with and without missing data for each outcome variable with regards to age at delivery, place of delivery, SBA, place of residence, SES and level of education.

### Ethical Considerations

Ethical approval was received from the Makerere University School of Public Health Research and Ethics Committee (MakSPH IRB 042) and the Uganda National Council of Science and Technology (UNCST SS2002). Informed consent was received from all participants in the surveys and data was handled so that anonymity was ensured. All respondents were told about the content and the purpose of the surveys, the expected time for participating, and that all information would be handled in such a way as to ensure anonymity. Data was stored on a password protected USB-memory after identifiers had been removed.

## Results

### Sociodemographic Characteristics of Respondents

There were 638 births amongst the participants of the SDG survey (Table 1, p. 14). The largest quintile was the poorest quintile (30%), whilst the least poor represented 10%. The majority of women had attained primary education (64%) and lived in rural areas (75%). Age at delivery ranged between 13 and 47 years with a mean age of 27 years. For the pregnancy and delivery survey, data collection was stopped when the required number was attained. A total of 411 women were visited, of whom 23 had out-migrated and two were unavailable for other reasons (Online Resource 2). The distribution of sociodemographic characteristics of the women in the pregnancy and delivery survey resembled those of all the women in the census survey.

**Table 1:** Sociodemographic characteristics of the women in the census and pregnancy and delivery survey

Sociodemographic characteristic		Census (N = 638)	Pregnancy and delivery survey (N = 386)
		% (n)	% (n)
Socio-economic status		N = 586	N = 358
	Poorest	30 (178)	31 (111)
	Poorer	22 (127)	24 (84)
	Poor	22 (129)	22 (80)
	Less poor	16 (92)	14 (50)
	Least poor	10 (60)	9 (33)
Level of education		N = 384	N = 241
	None	6 (24)	5 (11)
	Primary	64 (244)	68 (164)
	Secondary	26 (101)	24 (58)
	Vocational/Diploma	3 (13)	3 (8)
	Higher	0.5 (2)	0 (0)
Place of residence		N = 638	N = 386
	Peri-urban		
	Rural		
Birth outcome		N = 638	N = 386
	Single livebirth	96 (610)	96 (370)
	Twin live births	3 (21)	3 (13)
	Single stillbirths	1 (5)	1 (2)
	Twin stillbirths	0 (2)	0 (1)
		Years	Years
Age at delivery		N = 638	N = 386
	Min	13	13
	Mean (SD)	27 (7)	27 (7)
	Max	47	46

Information on health insurance, vaccinations, and postnatal visit were missing for a sub-set of women. However, no significant differences were found between women with and without missing data for all (Online Resource 3) except for health insurance coverage, which was significantly higher among women from households with higher SES (p-value < 0.01) and households located in peri-urban areas (p < 0.001) (Table S5).

### UHC for Essential Maternal Health Services

#### Service Coverage

**Fig. 1:**
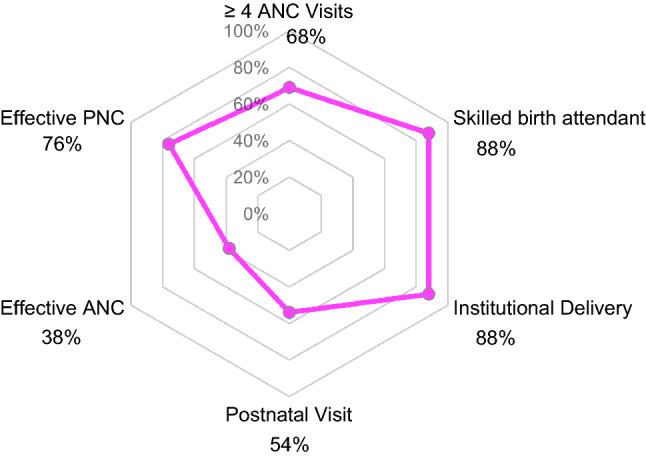
Extent of service coverage and effective coverage for maternal health services

Service coverage was highest for SBA and institutional delivery (88%) and lowest for a postnatal visit (54%) (Fig. 1, p. 19). Forty percent of women delivered in hospitals and a third in health centres (33%) (Table 2, p. 15). Most women (68%) reported at least four ANC visits.

**Table 2:** Service coverage and effective coverage

Service coverage	N	% (n)	Effective service coverage	N	% (n)
≥ 4 ANC visits	386	68 (264)	Effective coverage ANC	164	38 (62)
Skilled birth attendance (SBA)	638	88 (562)	Blood pressure	386	95 (365)
Institutional delivery	638	88 (563)	Weight	386	99 (383)
Delivery in hospital		40 (252)	Urine sample	386	51 (193)
Delivery in clinic		15 (98)	Blood pressure	386	98 (380)
Delivery in health centre		33 (213)	Tetanus vaccination	273	81 (222)
			Information about danger signs	386	92 (354)
Postnatal visit	274	54 (149)	Effective coverage PNC	164	76 (125)
			Health checked after delivery	386	83 (322)
			Newborn given polio and TB vaccination	273	89 (243)

Completion of four or more ANC visits was similar across the sub-groups (Table 3, p. 16). However, coverage of SBA was higher in women with higher SES (93% of the least poor). It was lower among women from rural areas than women from urban areas (87% vs 93%: p = 0.04). Similar distribution pattern was observed of institutional delivery. Of the poorest women and of women with no education, 83% delivered in a health facility, compared with 95% of the least poor and 100% of women with diploma/vocational or higher education. 86% of rural women and 94% of peri-urban women delivered in a health facility (p = 0.01). Coverage of a postnatal visit was higher among women with lowest SES (62% in the poorest vs 44% in the least poor quintile) and among women from rural areas (58%).

**Table 3:** Service coverage and effective coverage by socio-economic status (wealth quintile), level of education and place of residence

Variable	≥ 4 ANC visits	Effective ANC	Skilled birth attendance	Institutional delivery	Postnatal visit	Effective PNC
Wealth quintile	N = 358% (n)	N = 157% (n)	N = 586% (n)	N = 586% (n)	N = 261% (n)	N = 157% (n)
Poorest	61 (68)	35 (16)	81 (144)	83 (147)	62 (44)	70 (32)
Poorer	68 (57)	39 (16)	94 (119)	89 (113)	53 (32)	68 (27)
Poor	74 (59)	44 (16)	88 (114)	88 (113)	57 (35)	78 (29)
Less poor	70 (35)	32 ()	91 (84)	92 (85)	46 (20)	92 (23)
Least poor	70 (23)	44 (4)	93 (56)	95 (57)	44 (11)	89 (8)
P-value^a^	0.16	0.85^b^	0.01	0.01	0.07	0.13^b^
Level of education	N = 241% (n)	N = 107% (n)	N = 384% (n)	N = 384% (n)	N = 170% (n)	N = 106% (n)
None	73 (8)	0 (0)	92 (22)	83 (20)	42 (5)	80 (4)
Primary	65 (106)	40 (30)	85 (208)	85 (207)	66 (73)	74 (56)
Secondary	66 (38)	32 (7)	89 (90)	93 (94)	53 (21)	81 (17)
Diploma/Vocational	75 (6)	0 (0)	100 (13)	100 (13)	50 (3)	100 (4)
Higher	–	–	100 (2)	100 (2)	0 (0)	–
P-value^b^	0.93	0.16	0.55	0.13	0.17	0.83
Place of residence	N = 386% (n)	N = 164% (n)	N = 638% (n)	N = 638% (n)	N = 274% (n)	N = 164% (n)
Rural	69 (202)	40 (52)	87 (411)	86 (410)	58 (119)	72 (93)
Peri-urban	66 (62)	29 (10)	93 (151)	94 (153)	44 (30)	94 (32)
P-value^c^	0.34	0.32	0.04	0.01	0.04	0.01

^a^Chi-square (χ^2^) test of linear by linear association

^b^Fisher’s exact test

^c^Chi-square (χ^2^) test of independence

### Effective Coverage

Amongst the six ANC quality indicators, blood pressure and weight measurements, and blood sample were the most frequently reported (95–99%) (Table 2, p. 15). By contrast, 51% of women reported having a urine sample taken, leading to the overall ANC effective coverage of 38%. Over 80% of women had their health checked after delivery and almost 90% of neonates were given polio and TB vaccinations, resulting in the PNC effective coverage of 76%. Coverage of effective ANC was similar across the sub-groups (Table 3, p. 15). However, effective PNC coverage was lower among rural women than peri-urban women (72% vs 94%: p = 0.01) (Table 3, p. 16). There was no statistically significant difference between SES groups.

### Financial Coverage

Overall, 16% of the study population paid for ANC, whereas 91% paid for delivery services (Table 4, p. 17). Median out-of-pocket payments for caesarean section was slightly higher than for normal delivery (22,000 Shs vs 18,000 Shs). Health insurance coverage for maternal health services was almost nil (n = 1).

**Table 4:** Financial coverage for antenatal care and delivery service

Variable	N	% (n)
Paid for ANC	386	16 (62)
Paid for delivery	386	91 (351)
Out-of-pocket payments (Shillings)	N	Median (IQ Range)
< 4 ANC visits	386	0 (0–0)
≥ 4 ANC visits	386	0 (0–0))
Normal delivery	154	18,000 (7000–26,000)
Caesarean section	10	22,000 (12,000–50,000)
Delivery method unknown	222	18,000 (10,000–30,000)
Health Insurance	N	% (n)
Health insurance coverage	521	0 (1)

Out-of-pocket payments for delivery was higher in higher SES groups and higher levels of education and the differences were statistically significant (Table 5, p. 18). Median payment was highest in the less poor quintile (24,500 Shs), followed by the least poor and poor quintiles (20,000 Shs) and lowest in the poorest quintile (15,000 Shs) (p < 0.01). Median payment was higher for women with some education (p < 0.01). However, the correlation between higher out-of-pocket payments and higher level of education was 0.20. Median payment was significantly higher among women from peri-urban areas than for women from rural areas (24,500 Shs vs 16,500 Shs; p < 0.001).

**Table 5:** Financial coverage by socio-economic status (wealth quintile), level of education and place of residence

Variable	Out-of-pocket payments ANC (Shillings)	Out-of-pocket payments delivery (Shillings)	Health insurance coverage
Wealth quintile	N = 358 Median (IQ range)	N = 358 Median (IQ range)	N = 488% (n)
Poorest	0 (0–0)	15,000 (5000–19,000)	0 (0)
Poorer	0 (0–0)	16,500 (5000–25,000)	0 (0)
Poor	0 (0–0)	20,000 (10,000–27,500)	0 (0)
Less poor	0 (0–0)	24,500 (10,000–40,000)	0 (0)
Least poor	0 (0–0)	20,000 (15,000–37,000)	0 (1)
P-value^a^	n/a	p < 0.01	n/a
Level of education	N = 241 Median (IQ range)	N = 241 Median (IQ range)	N = 334% (n)
None	0 (0–4000)	12,000 (6000–16,000)	0 (0)
Primary	0 (0–0)	18,000 (10,000–25,000)	0 (0)
Secondary	0 (0–0)	20,000 (10,000–35,000)	0 (0)
Diploma/Vocational	0 (0–0)	17,500 (10,000–300,000)	0 (0)
Correlation Coefficient^b^		0.20	
P-value^b^	n/a	p < 0.01	n/a
Place of residence	N = 386 Median (IQ range)	N = 386 Median (IQ range)	N = 521% (n)
Rural	0 (0–0)	16,500 (7000–25,000)	0 (1)
Peri-urban	0 (0–0)	24,500 (15,000–38,000)	0 (0)
P-value^c^	n/a	p < 0.001	n/a

^a^Kruskal-Wallis H test

^b^Spearman rank correlation

^c^Mann-Whitney U test

## Discussion

This study measured the extent of service, effective and financial coverage for routine maternal health services, using health and demographic surveillance site data. The results indicate near universal service coverage for institutional delivery and SBA, however, gaps exist in both service and effective coverage for ANC and PNC. Health insurance coverage was close to non-existing. The majority of women paid for delivery service (91%) with the median payment for normal delivery being 18,000 Shs and that for caesarean section delivery being 22,000 Shs. SBA and institutional delivery were more common among women with higher SES and among women from peri-urban areas. By contrast, a postnatal visit was more common among rural women. Women from peri-urban areas, with higher SES and with higher education made higher out-of-pocket payments.

Coverages of the Maternal health service and effective coverage in the current study were higher compared with the most recent DHS data (Demographic & Health Survey program, 2016). One potential explanation is that a number of maternal and new-born health interventions have been implemented within the relatively small area of IMHDSS (Lawn et al., 2015) Therefore, women in IMHDSS could have a higher awareness of recommended health services and in effect utilise them to a higher extent compared to women outside the surveillance site. Despite this, quality of ANC remains low, as a previous study reported co-coverage of four or more ANC visits and seven indicators of care content of 10% in 2011 (Benova et al., 2018).

Health insurance coverage was lacking, in line with the low estimations of national coverage, especially in rural areas (Uganda Bureau of Statistics, 2018). The payments for delivery services were both more common and higher compared with ANC payments. Out-of-pocket payments for the delivery service are likely to have a negative impact on household welfare. Similar to findings from previous studies from low- and middle-income countries (Kruk et al., 2016), this study’s findings indicate that women delivering by caesarean section, requiring in-hospital stay, results in high out-of-pocket payments. Although the Ugandan health system officially provides maternal health services free of charge, the implementation of the policy appears poor. This has been described in other countries where user-fees have officially been removed (Dalinjong et al., 2017), highlighting that measuring payments at the point of service delivery is important.

Similar to previous studies on service coverage of reproductive, maternal, newborn and child health (Boerma et al., 2018), maternal health services provided at community level had higher coverage among women from poorer households, while delivery services provided at healthcare facilities had higher coverage among women from wealthier households. Similarly, Waiswa, Pariyo, et al. (2015) found that coverage of a postnatal visit by community health workers was “pro-poor” having higher concentration among poor women in IMHDSS. Despite this, the poor and rural women received lower quality PNC.

Strengths of this study is that we applied the WHO/WB’s framework for monitoring UHC, including the recommended service coverage indicators of pregnancy and delivery care. Eight content indicators of ANC and PNC were analysed, capturing quality aspects (Table 2, p. 15). Additionally, we estimated financial coverage (protection) and lack thereof for these services by extent of out-of-pocket payments and health insurance coverage. An advantage in asking the women who received the healthcare services is that it measures the output of the health delivery system. Using data from a rural poor setting in Uganda, the study provides valuable information to achieve UHC for its entire population. The study highlighted where coverage gaps exist, the scope of out-of-pocket payments for maternal health services, as well as measurements of the contents of care to indicate the quality of the health services.

Our study had limitations. Effective coverage is often measured by incorporating the health service coverage indicator and the quality indicator(s) (Ng et al., 2014). However, in this study, service coverage and effective coverage were analysed separately. Because the PNC visit measured a VHT visit on community level and the content of PNC included healthcare content related to the time immediately after childbirth, these indicators didn’t allow for an aggregate coverage measure. Future attempts at computing effective coverage utilising different data sources could explore analytical techniques for linking the data, such as ecological linkage using geocoordinates of health facilities or other innovative techniques (Amouzou et al., 2019).

The large amount of missing data for tetanus, polio and TB vaccinations and postnatal visit possibly led to the lack of statistical power for the equity analysis. However, since missingness was random, conclusions can still be drawn on the proportions of total coverage. Because missing data for health insurance coverage was significantly higher among women with higher SES and peri-urban women, there is a possibility that health insurance coverage is underestimated.

The ability to measure healthcare quality for maternal health services using household survey data is limited, particularly for the quality of delivery care, which was not estimated in this study. Surveys and census like IMHDSS rely on participants’ recall at the point of healthcare contact. Participants’ health literacy or recall may be limited, resulting in an inability to accurately account for the content of care received. An alternative approach could be to analyse facility-based records. Thus, strengthening health facility data and investing in country health information systems is warranted (Marchant et al., 2019).

### Implications for Policy and Future Research

Coverage of all six ANC indicators represented the lowest effective coverage indicator. Assuring women receive full ANC content is important for detection and management of morbidity during pregnancy (World Health Organization, 2016). Thus, the resources, readiness, and procedures at health facilities for ANC provision should be investigated further.

To what extent the observed differences between women’s out-of-pocket payments during childbirth with regard to SES, place of residence and education can be explained by the women attending different healthcare providers or receiving different contents of care warrants further investigation. Waiswa et al. (2015) found that public health facility delivery was more common than private health facility delivery within IMHDSS and that women delivering in private health facilities was associated with lower SES and less education. Finally, to determine the effect of out-of-pocket payments on households, more data on household income and the content of payments are needed.

Measures of UHC should incorporate all dimensions of the concept. Computing a single UHC composite indicator has been attempted (Prinja et al., 2017). Such a composite indicator can offer a quick illustration of health system performance and allow comparison (Prinja et al., 2017). However, monitoring separate indicators, as done in this study, is likely more relevant to inform policy makers where coverage gaps exist (Prinja et al., 2017).

To further develop UHC measurements using HDSS data, some challenges and considerations are recognised. Estimating financial risk protection requires household healthcare expenditure data, involving resource intensive data collection and careful methodological choices (Saksena et al., 2014), such as thresholds for catastrophic healthcare expenditure (Hsu et al., 2018). Improved facility data is needed to obtain a quality adjusted coverage (Amouzou et al., 2019; Marchant et al., 2019). Future research should explore the potential to utilise routinely collected HDSS data, additional household or individual surveys, in combination with linked health records. By continuously collecting data at surveillance sites, an opportunity exists to track UHC at district level.

## Conclusion

Maternal health service coverage in eastern Ugandan is not universal. The largest coverage gap was found in PNC and ANC, particularly effective ANC. While coverage of SBA and institutional delivery were higher among wealthier and urban women, PNC service coverage was higher among rural residents but with poorer care quality. Financial coverage was close to non-existent with higher out-of-pocket payments made by women of higher socio-economic status, higher education and peri-urban residence. Exploiting health facility data to measure quality of delivery services and assessing UHC by type of healthcare provider are areas of future research.

## Supplementary Information

Below is the link to the electronic supplementary material.

Supplementary file1 (DOCX 18 kb)

Supplementary file2 (DOCX 62 kb)

Supplementary file3 (DOCX 37 kb)

## Data Availability

Core surveillance data have been shared through the iShare initiative of INDEPTH and are accessible online.

## Code Availability

Available upon request.
