# Supplementary material for: The Extent of Universal Health Coverage for Maternal Health Services in Eastern Uganda: A Cross Sectional Study
Source: Matern Child Health J. 2021 Dec 30;26(3):632–41. doi: 10.1007/s10995-021-03357-3 (PMC8917020; doi:10.1007/s10995-021-03357-3)
Supplement: Supplementary file 1 — Supplementary file1 (DOCX 18 kb) [file 10995_2021_3357_MOESM1_ESM.docx]

**Supplementary Material.** Pregnancy and delivery survey

This is a follow-up from previous round visits, and I would like to ask you some questions regarding your childbirth of (Name) in 2017 and its pregnancy period. This will only take a few minutes. If you do not want to answer, you don’t have to, and you can opt out at any time you want, but your participation in this study will be of great value. This work is part of the HDSS and it shall be used for research purposes only. There are no direct benefits or risks involved in participating in this study. The information can be useful in putting up strategies to improve maternal and newborn care in Uganda. All information will be handled with confidentiality and anonymity will be ensured.

Do you want to participate?

Yes No

**1.** How many times did you go for ANC during the pregnancy for which you gave birth to (Name) in 2017?

None= 0 1= 1 2= 2 3= 3 4= 4 more than 4= 5

If the answer to Q1 is 1 or more then ask the following questions (2 and 3).

**2.** Did you pay any money to cater for treatment and/or other services received during the ANC visit(s); like consultation fee, payment for drugs or tests?

1= Yes 2= No

If yes.

How much in total?

Amount (Shs)…………………………………….

**3.** As part of your antenatal care during this pregnancy, were any of the following done at least once:

Was your blood pressure measured? 1= Yes 2= No 3= Don’t remember

Did you give a urine sample? 1= Yes 2= No 3= Don’t remember

Did you give a blood sample? 1= Yes 2= No 3= Don’t remember

Was your weight measured? 1= Yes 2= No 3= Don’t remember

**3b.** And were you told by a health worker about danger signs that might indicate problems with the pregnancy? (e.g. abdominal pain, bleeding, severe headache with blurred vision) 1= Yes 2= No 3= Don’t remember

**4.** Approximately how much in total did you pay your delivery service provider for your delivery of (Name) in 2017? (for admission fee, drugs/supplies, operating theatre, food, tests and in-patient stay) [If no amount spent, put zero(0)]

Amount (Shs)…………………………………….

**5.** After you had delivered, did a nurse/midwife/doctor check on your health while you were still in the health facility/the place you delivered?

1= Yes 2= No 3= Don’t remember
