# Supplementary material for: The Extent of Universal Health Coverage for Maternal Health Services in Eastern Uganda: A Cross Sectional Study
Source: Matern Child Health J. 2021 Dec 30;26(3):632–41. doi: 10.1007/s10995-021-03357-3 (PMC8917020; doi:10.1007/s10995-021-03357-3)
Supplement: Supplementary file 2 — Supplementary file2 (DOCX 62 kb) [file 10995_2021_3357_MOESM2_ESM.docx]

**Table SI**. Outcome variables, with respective indicators and their source.

| **Outcome variable** | **Indicator** | **Source** |
| --- | --- | --- |
| Service coverage | Number of antenatal care visits | Pregnancy & delivery survey |
|  | Place of delivery | Census |
|  | Type of birth assistant | Census |
|  | Postnatal visit received | Census |
| Effective ANC | Blood pressure measured during ANC visit | Pregnancy & delivery survey |
|  | Urine sample taken during ANC visit | Pregnancy & delivery survey |
|  | Blood sample given during ANC visit | Pregnancy & delivery survey |
|  | Weight measured during ANC visit | Pregnancy & delivery survey |
|  | Information about danger signs received | Pregnancy & delivery survey |
|  | Tetanus vaccination during pregnancy | Census |
| Effective PNC | Post-delivery health check by nurse/midwife/doctor | Pregnancy & delivery survey |
|  | Polio & Tb vaccinations received by newborn | Census |
| Financial coverage | Health insurance coverage | SDG survey |
|  | Out-of-pocket payment for antenatal care | Pregnancy & delivery survey |
|  | Out-of-pocket payment for delivery | Pregnancy & delivery survey |
|  | Wealth quintile | Census |
| Covariate equity analysis | Highest level of education attained | Census |
|  | Place of residence | Census |
| Descriptive variables | Type of delivery method | Census |
|  | Birth outcome | Census |
|  | Age at delivery | Census |

**Figure S1.** Flow chart of data included in the study.

23 mothers had out-migrated, 2 were unavailable and 38 were not visited

364missing

18,634 households in the HDSS

365 missing

117 missing

365 missing

364 missing

274 births included for coverage of postnatal visit

273 births included for coverage of tetanus vaccination

273 births included for coverage of polio and Tb vaccination

521 births included for health insurance for maternal health services

All 638 births analysed for coverage of SBA and institutional delivery

Interview conducted with 386 women on number of ANC visits, 5 content indicators of ANC, ANC costs, post-delivery health check and delivery costs

23 mothers had out-migrated, 2 were unavailable and 38 were not visited

449 women randomly sampled for the pregnancy survey

638 births occurred in 2017

5,500 households randomly sampled for SDG survey

Clara Lindberg

23/05/2019

**25**

Clara Lindberg

23/05/2019

**25**
