# Supplementary material for: The Extent of Universal Health Coverage for Maternal Health Services in Eastern Uganda: A Cross Sectional Study
Source: Matern Child Health J. 2021 Dec 30;26(3):632–41. doi: 10.1007/s10995-021-03357-3 (PMC8917020; doi:10.1007/s10995-021-03357-3)
Supplement: Supplementary file 3 — Supplementary file3 (DOCX 37 kb) [file 10995_2021_3357_MOESM3_ESM.docx]

**Table S2.** Sensitivity analysis missing data: tetanus vaccination received during pregnancy.

|  | **Missing data** | **No missing data** | **P-value** |
| --- | --- | --- | --- |
|  | **N=365** | **N=273** |  |
| **Age group** | **% (n)** | **% (n)** |  |
| <15 | 1 (2) | 0 (1) | 0.64^a^ |
| 15-20 | 19 (68) | 21 (56) |  |
| 21-25 | 28 (103) | 26 (70) |  |
| 26-30 | 25 (91) | 24 (66) |  |
| 31-35 | 17 (62) | 14 (39) |  |
| 36-40 | 7 (27) | 13 (35) |  |
| 41-45 | 3 (12) | 1 (3) |  |
| >45 | 0 (0) | 1 (3) |  |
| **Place of delivery** |  |  |  |
| Hospital | 38 (137) | 42 (115) | 0.42^b^ |
| Health centre | 34 (123) | 33 (90) |  |
| Clinic | 17 (61) | 14 (37) |  |
| TBA | 4 (16) | 4 (10) |  |
| Home | 7 (24) | 8 (21) |  |
| On the way to facility | 1 (4) | 0 (0) |  |
| Institutional delivery | 88 (321) | 89 (242) | 0.79^c^ |
| Non-institutional delivery | 12 (44) | 11 (31) |  |
| **Birth attendant** |  |  |  |
| Skilled birth attendant | 87 | 90 | 0.17^c^ |
| Non-skilled birth attendant | 12 | 11 |  |
| **Place of residence** |  |  |  |
| Rural | 74 (271) | 75 (204) | 0.89^c^ |
| Peri-urban | 26 (94) | 25 (69) |  |
| **Socio-economic status** |  |  |  |
| Poorest | 33 (107) | 27 (71) | 0.45^d^ |
| Poorer | 21 (67) | 23 (60) |  |
| Poor | 21 (69) | 23 (60) |  |
| Less poor | 15 (48) | 17 (44) |  |
| Least poor | 11 (35) | 10 (25) |  |
| **Level of education** |  |  |  |
| None | 6 (12) | 7 (12) | 0.83^b^ |
| Primary | 62 (133) | 65 (111) |  |
| Secondary | 29 (61) | 24 (40) |  |
| Diploma/vocational | 3 (7) | 4 (6) |  |
| Higher | 1 (1) | 1 (1) |  |

^a^ Independent T-test

^b^ Fisher’s exact test

^c^ Chi-square (χ^2^) test of independence

^d^ Chi-square (χ^2^) test of linear by linear association

**Table S3.** Sensitivity analysis missing data: polio and Tb vaccination given to newborn child.

|  | **Missing data** | **No missing data** | **P-value** |
| --- | --- | --- | --- |
|  | **N=365** | **N=273** |  |
| **Age group** | **% (n)** | **% (n)** |  |
| <15 | 1 (2) | 0 (1) | 0.89^a^ |
| 15-20 | 18 (67) | 21 (57) |  |
| 21-25 | 28 (103) | 26 (70) |  |
| 26-30 | 25 (91) | 24 (66) |  |
| 31-35 | 17 (62) | 14 (39) |  |
| 36-40 | 7 (27) | 13 (35) |  |
| 41-45 | 4 (13) | 1 (2) |  |
| >45 | 0 (0) | 1 (3) |  |
| **Place of delivery** |  |  |  |
| Hospital | 38 (137) | 42 (115) | 0.50^b^ |
| Health centre | 34 (123) | 33 (90) |  |
| Clinic | 16 (60) | 14 (38) |  |
| TBA | 4 (16) | 4 (10) |  |
| Home | 7 (25) | 7 (20) |  |
| On the way to facility | 1 (4) | 0 (0) |  |
| Institutional delivery | 88 (320) | 89 (243) | 0.60^c^ |
| Non-institutional delivery | 12 (45) | 11 (30) |  |
| **Birth attendant** |  |  |  |
| Skilled birth attendant | 87 (316) | 90 (246) | 0.17^c^ |
| Non-skilled birth attendant | 13 (49) | 10 (27) |  |
| **Place of residence** |  |  |  |
| Rural | 74 (271) | 75 (204) | 0.89^c^ |
| Peri-urban | 26 (94) | 25 (69) |  |
| **Socio-economic status** |  |  |  |
| Poorest | 33 (107) | 27 (71) | 0.41^d^ |
| Poorer | 21 (68) | 23 (59) |  |
| Poor | 21 (68) | 24 (61) |  |
| Less poor | 15 (48) | 17 (44) |  |
| Least poor | 11 (35) | 10 (25) |  |
| **Level of education** |  |  |  |
| None | 6 (12) | 7 (12) | 0.76^b^ |
| Primary | 62 (133) | 66 (111) |  |
| Secondary | 29 (62) | 23 (39) |  |
| Diploma/vocational | 3 (7) | 4 (6) |  |
| Higher | 1 (1) | 1 (1) |  |

^a^ Independent T-test

^b^ Fisher’s exact test

^c^ Chi-square (χ^2^) test of independence

^d^ Chi-square (χ^2^) test of linear by linear association

**Table S4.** Sensitivity analysis missing data: postnatal visit by a VHT.

|  | **Missing data** | **No missing data** | **P-value** |
| --- | --- | --- | --- |
|  | **N=364** | **N=274** |  |
| **Age group** | **% (n)** | **% (n)** |  |
| <15 | 1 (2) | 0 (1) | 0.73^a^ |
| 15-20 | 18 (67) | 21 (57) |  |
| 21-25 | 28 (103) | 26 (70) |  |
| 26-30 | 25 (91) | 24 (66) |  |
| 31-35 | 17 (62) | 14 (39) |  |
| 36-40 | 7 (27) | 13 (35) |  |
| 41-45 | 4 (12) | 1 (3) |  |
| >45 | 0 (0) | 1 (3) |  |
| **Place of delivery** |  |  |  |
| Hospital | 38 (137) | 42 (115) | 0.47^b^ |
| Health centre | 34 (123) | 33 (90) |  |
| Clinic | 17 (60) | 14 (38) |  |
| TBA | 4 (16) | 4 (10) |  |
| Home | 7 (24) | 8 (21) |  |
| On the way to facility | 1 (4) | 0 (0) |  |
| Institutional delivery | 88 (320) | 89 (243) | 0.76^c^ |
| Non-institutional delivery | 12 (44) | 11 (31) |  |
| **Birth attendant** |  |  |  |
| Skilled birth attendant | 87 (315) | 90 (247) | 0.16^c^ |
| Non-skilled birth attendant | 14 (49) | 10 (27) |  |
| **Place of residence** |  |  |  |
| Rural | 74 (270) | 75 (205) | 0.85^c^ |
| Peri-urban | 26 (94) | 25 (69) |  |
| **Socio-economic status** |  |  |  |
| Poorest | 33 (107) | 27 (71) | 0.43^d^ |
| Poorer | 21 (67) | 23 (60) |  |
| Poor | 21 (68) | 23 (61) |  |
| Less poor | 15 (48) | 17 (44) |  |
| Least poor | 11 (35) | 10 (25) |  |
| **Level of education** |  |  |  |
| None | 6 (12) | 7 (12) | 0.83^b^ |
| Primary | 62 (133) | 65 (111) |  |
| Secondary | 29 (61) | 24 (40) |  |
| Diploma/vocational | 3 (7) | 4 (6) |  |
| Higher | 1 (1) | 1 (1) |  |

^a^ Independent T-test

^b^ Fisher’s exact test

^c^ Chi-square (χ^2^) test of independence

^d^ Chi-square (χ^2^) test of linear by linear association

**Table S5.** Sensitivity analysis missing data: health insurance coverage.

|  | **Missing data** | **No missing data** | **P-value** |
| --- | --- | --- | --- |
|  | **N=117** | **N=521** |  |
| **Age group** | **% (n)** | **% (n)** |  |
| <15 | 0 (0) | 1 (3) | 0.14^a^ |
| 15-20 | 21 (25) | 19 (99) |  |
| 21-25 | 31 (36) | 26 (137) |  |
| 26-30 | 21 (24) | 26 (133) |  |
| 31-35 | 21 (25) | 15 (76) |  |
| 36-40 | 4 (5) | 11 (57) |  |
| 41-45 | 2 (2) | 3 (13) |  |
| >45 | 0 (0) | 1 (3) |  |
| **Place of delivery** |  |  |  |
| Hospital | 47 (55) | 38 (197) | 0.57^b^ |
| Health centre | 30 (35) | 34 (178) |  |
| Clinic | 14 (16) | 16 (82) |  |
| TBA | 4 (5) | 4 (21) |  |
| Home | 5 (6) | 8 (39) |  |
| On the way to facility | 0 (0) | 1 (4) |  |
| Institutional delivery | 91 (106) | 88 (457) | 0.38^c^ |
| Non-institutional delivery | 9 (11) | 12 (64) |  |
| **Birth attendant** |  |  |  |
| Skilled birth attendant | 87 (106) | 90 (456) | 0.35^c^ |
| Non-skilled birth attendant | 14 (11) | 10 (65) |  |
| **Place of residence** |  |  |  |
| Rural | 56 (66) | 79 (409) | <0.001^c^ |
| Peri-urban | 44 (51) | 22 (112) |  |
| **Socio-economic status** |  |  |  |
| Poorest | 20 (20) | 32 (158) | <0.01^d^ |
| Poorer | 24 (23) | 21 (104) |  |
| Poor | 18 (18) | 23 (111) |  |
| Less poor | 21 (21) | 15 (71) |  |
| Least poor | 16 (16) | 9 (44) |  |
| **Level of education** |  |  |  |
| None | 2 (1) | 7 (23) | 0.21^b^ |
| Primary | 66 (33) | 63 (211) |  |
| Secondary | 24 (12) | 27 (89) |  |
| Diploma/vocational | 6 (3) | 3 (10) |  |
| Higher | 2 (1) | 0 (1) |  |

^a^ Independent T-test

^b^ Fisher’s exact test

^c^ Chi-square (χ^2^) test of independence

^d^ Chi-square (χ^2^) test of linear by linear association
